# Vasorelaxant Activities and its Underlying Mechanisms of Magnolia Volatile Oil on Rat Thoracic Aorta Based on Network Pharmacology

**DOI:** 10.3389/fphar.2022.812716

**Published:** 2022-02-28

**Authors:** Jin-Feng Xu, Jia Xia, Yan Wan, Yu Yang, Jiao-Jiao Wu, Cheng Peng, Hui Ao

**Affiliations:** ^1^ Key Laboratory of Southwestern Chinese Medicine Resources, Chengdu University of Traditional Chinese Medicine, Chengdu, China; ^2^ Innovative Institute of Chinese Medicine and Pharmacy, Chengdu University of Traditional Chinese Medicine, Chengdu, China

**Keywords:** Magnolia volatile oil, network pharmacology, vasorelaxant effect, endothelial dependence and -independence, hypertension

## Abstract

**Objective:** Magnolia volatile oil (MVO) is a mixture mainly containing eudesmol and its isomers. This study was to investigate the vasorelaxant effects and the underlying mechanism of MVO in rat thoracic aortas.

**Method:** The present study combined gas chromatography–mass spectrometry (GC-MS) and network pharmacology analysis with *in vitro* experiments to clarify the mechanisms of MVO against vessel contraction. A compound–target network, compound–target–disease network, protein–protein interaction network, compound–target–pathway network, gene ontology, and pathway enrichment for hypertension were applied to identify the potential active compounds, drug targets, and pathways. Additionally, the thoracic aortic rings with or without endothelium were prepared to explore the underlying mechanisms. The roles of the PI3K-Akt-NO pathways, neuroreceptors, K^+^ channels, and Ca^2+^ channels on the vasorelaxant effects of MVO were evaluated through the rat thoracic aortic rings.

**Results:** A total of 29 compounds were found in MVO, which were identified by GC-MS, of which 21 compounds with a content of more than 0.1% were selected for further analysis. The network pharmacology research predicted that beta-caryophyllene, palmitic acid, and (+)-β-selinene might act as the effective ingredients of MVO for the treatment of hypertension. Several hot targets, mainly involving TNF, CHRM1, ACE, IL10, PTGS2, REN, and F2, and pivotal pathways, such as the neuroactive ligand–receptor interaction, the calcium signaling pathway, and the PI3K-Akt signaling, were responsible for the vasorelaxant effect of MVO. As expected, MVO exerted a vasorelaxant effect on the aortic rings pre-contracted by KCl and phenylephrine in an endothelium-dependent and non-endothelium-dependent manner. Importantly, a pre-incubation with indomethacin (Indo), *N*-nitro-*L*-arginine methyl ester, methylene blue, wortmannin, and atropine sulfate as well as 4-aminopyridione diminished MVO-induced vasorelaxation, suggesting that the activation of the PI3K-Akt-NO pathway and K_V_ channel were involved in the vasorelaxant effect of MVO, which was consistent with the results of the Kyoto Encyclopedia of Genes and the Genomes. Additionally, MVO could significantly inhibit Ca^2+^ influx resulting in the contraction of aortic rings, revealing that the inhibition of the calcium signaling pathway exactly participated in the vasorelaxant activity of MVO as predicted by network pharmacology.

**Conclusion:** MVO might be a potent treatment of diseases with vascular dysfunction like hypertension. The underlying mechanisms were related to the PI3K-Akt-NO pathway, K_V_ pathway, as well as Ca^2+^ channel, which were predicted by the network pharmacology and verified by the experiments *in vitro.* This study based on network pharmacology provided experimental support for the clinical application of MVO in the treatment of hypertension and afforded a novel research method to explore the activity and mechanism of traditional Chinese medicine.

## 1 Introduction

Hypertension is a pivotal cause for the occurrence of cardiac-cerebral vascular diseases (CCVDs) relevant to complications including stroke, kidney damage, dementia, blindness, myocardial infarction, cancer, and heart failure (August, 2004; Sengul et al., 2016; Volpe et al., 2018; Beaney et al., 2020). It is usually accompanied with pathological characters by the reduction of vasorelaxant capacities and the enhancement of vasocontractile reactions responsible for the lack of the blood supply to important organs and tissues (Kizhakekuttu and Widlansky, 2010; Kidoguchi et al., 2021), which is a serious threat to human health, especially for cardiovascular patients. At present, merely 34% of hypertensive patients manage their blood pressure satisfactorily, and their intake of anti-hypertensive medicines is restricted because of their serious adverse reaction to the medicines and the medicines’ high cost, especially after they are given continuously to the patients for a prolonged period (Akhtar et al., 2016; Maranta et al., 2017 ). Thereby, the increasing number of issues prompted the search for alternative and complementary therapies for better treatment of hypertension, which are of higher efficiency, lower cost, and minimal toxicity.

*Magnolia officinalis* Rehd. et Wils. contains phenylpropanoids, lignans, glycosides, alkaloids, flavonoids, essential oil, terpenoids, etc. (Kelm and Nair, 2000), and is a medicinal herb that has been used in the treatment of hyperglycemia (Sun et al., 2015), epilepsy (Chen et al., 2011), dementia (Lee et al., 2012), tumor (Youn et al., 2013), anxiety (Han et al., 2011), and so on. At present, not only the extractions derived from the leaf of *M. officinalis* Rehd. et Wils. but also the main active ingredient (β-eudesmol) in MVO (Obara et al., 2002; Baek, 2004; Lim and Kee, 2005; Yang et al., 2012) are identified to have a vasorelaxant activity. However, little was known about the effect on the potential vascular activity and the mechanisms of oils extracted from the bark of *M. officinalis* Rehd. et Wils. As MVO is a complicated mixture, it is difficult to elucidate its active ingredients and therapeutic mechanisms, which is likely to be related to its multiple pharmacological activities, biological targets, and pathways. Fortunately, network pharmacology, an innovative and effective approach, is in the light of computational systems pharmacology, which exactly decodes the connections among the herbs, targets, pathways, and diseases at a comprehensive and systematic level (Li, 2016; Yu and Wang, 2016; Luo et al., 2020) and has been successfully applied to study the complex mechanisms of traditional Chinese medicine (TCM) (He et al., 2019). Thereby, the chemical constituents of MVO were analyzed by gas chromatography–mass spectrometry (GC-MS) and the combination of prediction of network pharmacology and verification of rat thoracic aorta was applied to explore the vasorelaxant activity and the underlying mechanisms of MVO. The experiment method was convenient, stable, and reliable, which has been proved by a number of researches (Ferreira et al., 2007; Wang et al., 2015).

In short, to our knowledge, the present study was the first report to holistically reveal the correlation between the mechanisms and the vasorelaxant effect of MVO treatment *via* the combination of network pharmacology prediction and experimental verification, which provided the scientific evidences for the clinical applications of MVO and created a novel direction to explore the activities and mechanisms of TCM.

## 2 Materials

### 2.1 Herbs and Reagents

The bark of *M. officinalis* Rehd. et Wils. was bought from the Chengdu International Trade city of Hehuachi TCM market, which was identified by Chen, an associate professor of the Chengdu University of Traditional Chinese Medicine, as the dry rind of *M. officinalis* Rehd. et Wils. *M. officinalis* Rehd. et Wils. in the experiment was a mixture of equal proportions from seven different habitats, mainly including Chengdu (Sichuan Province, China), Guiyang (Guizhou Province, China), Anhua (Hunan Province, China), Enshi (Hubei Province, China), Shaoxing (Zhejiang Province, China), Daoxian (Hunan Province, China), and Liu’an (Anhui Province, China).

*N*-hexane and anhydrous sodium sulfate were bought from Sinopharm Chemical Reagent Co., Ltd. Dimethyl sulfoxide was obtained from Chengdu Kelon Chemical Reagent Factory.

### 2.2 Experimental Animals

Male Sprague–Dawley (SD) rats weighing 260–320 g were provided by the Chengdu Dossy Experimental Animals Co., Ltd. (Chengdu, China) [Sichuan Province Animal Use Certificate No. SCXK (Chuan) 2013-15]. All animal care and experimental protocols followed the Animal Management Rules of the Ministry of Health of China. The rats were housed at 25°C, with a 12 h light/dark cycle and with free access to water and food for 5–7 days before the experiments.

## 3 Methods

### 3.1 The Extraction and Analysis of MVO

#### 3.1.1 Extraction and Preparation of MVO

The bark of *M. officinalis* Rehd. et Wils. was smashed. About 500 g of the sample was added into the original bottom flask (10,000 ml) with 4,000 ml of ultrapure water. MVO samples were prepared by water distillation for l5 h until the oil quantity in the extractor did not increase, obtained from the condenser, and dried over anhydrous sodium sulfate, and finally stored in a sealed and dark glass bottle at 4°C prior to GC-MS analysis.

The calculation formula of MVO extraction rate is as follows: ER = V/m × 100%. ER is the volatile oil extraction rate (%); V is the MVO volume (ml); and m is the weight (g) of the bark of *M. officinalis* Rehd. Et Wils.

#### 3.1.2 GC-MS Analysis of MVO

GC-MS analysis was performed on an Agilent Technologies apparatus 7890A-5975C with HP-INNOWAX MS capillary column (30 m × 250 μm × 0.25 μm). The following oven temperature program was initiated at 60°C, held for 5 min, then increased at the rate of 10°C min^− 1^–120°C, held for 0 min, next increased at the rate of 2°C min^− 1^–185°C, held for 3 min, and finally increased at the rate of 8°C·min^− 1^–250°C. The carrier gas was helium (He) at a constant flow of 1 ml min^− 1^. The ion source was kept at 230°C. The splitting ratio was 10:1. The spectrometer operated at 70 eV with the scan from (m/z) 35–550 Da.

### 3.2 Prediction of the Vasorelaxant Activities and The Underlying Mechanisms of MVO Based on Network Pharmacology

#### 3.2.1 Identification of Active Compounds and Prediction of Corresponding Targets of MVO

According to the results of GC-MS, the ingredients with a content of more than 0.1% were screened as the effective ingredients of MVO for in-depth analysis. In the following description, we called those compounds of MVO “the active compounds of MVO.” The Traditional Chinese Medicine Systems Pharmacology database (TCMSP, https://tcmsp-e.com/) was introduced to discover the targets of those compounds. Then, the target names were inputted into the UniProt database (http://www.uniprot.org/) with the species selected as “Homo sapiens,” and the gene symbols of the targets were obtained from the UniProt database. In the following description, we called this part of the gene set “the targets of MVO.”

#### 3.2.2 Collection of Targets Related to Hypertension

To ensure the comprehensiveness of hypertension-related genes collected, three databases were selected to search targets. More concretely, the keywords “Hypertension” was inputted in Online Mendelian Inheritance in Man (OMIM, https://omim.org/), GeneCards (http://www.genecards.org/), and Therapeutic Target Database (TTD, http://db.idrblab.net/ttd/) to collect the hypertension-related targets. Afterwards, the obtained targets were also sent to the UniProt database for normalization. Finally, hypertension-related targets were discovered from the above databases after deleting redundancy. In the following description, we called this part of the gene set “genes related to hypertension.”

A Venn diagram was drawn using an online website (http://bioinformatics.psb.ugent.be/Webtools/Venn/) to get the same targets between the genes related to hypertension and the target of MVO, which might be the potential targets of MVO in hypertension treatment. In the following description, we called this part of the gene set “overlapping targets of MVO and hypertension.”

#### 3.2.3 Construction of Protein–Protein Interaction Network and Screening of Hub Targets

To figure out the direct and indirect overlapping targets of MVO and hypertension interaction and explore the genes associated with them, the Search Tool for the Retrieval of Interacting Genes (STRING) 11.0 database (https://string-db.org/) was employed (Chen et al., 2021). Based on the overlapping targets of MVO and hypertension, we constructed the protein–protein interaction (PPI) network by using the STRING 11.0 database with the species limited to “Homo sapiens” and confidence score >0.4. The topological properties of the PPI network, including the degree, average shortest path length, betweenness centrality, and closeness centrality, were determined in the Cytoscape 3.8.2 (https://cytoscape.org/; version 3.8.2). Additionally, the degree value was used to select the putative targets for experimental verification.

#### 3.2.4 Gene Ontology and Pathway Enrichment Analysis

For a better demonstration of the potential biological processes and pathways of MVO in the treatment of hypertension in this study, we utilized the Metascape database (https://metascape.org/gp/index.html#/main/step1) to explore the gene ontology (GO) function enrichment analysis, the Kyoto Encyclopedia of Genes and the Genomes (KEGG) pathway enrichment analysis (Yingyao Zhou et al., 2019). The GO terms and pathway terms with a *P*-value <0.05 were considered as significant enrichment entries.

#### 3.2.5 Network Construction and Analysis

In this study, multiple networks were established to visualize and analyze the complicated interconnection of compounds, targets, and diseases in Cytoscape 3.8.2 software. Based on the above results, the compound–target (C–T) network, the compound–target–disease (C–T–D) network, the PPI network, and the compound–target–pathway (C–T–P) network were constructed in Cytoscape 3.8.2 software. In these networks, the nodes of different colors and shapes represented different active compounds, potential targets, or signal pathways, and the edges represented the connections between the nodes.

### 3.3 Verification of the Vasorelaxation Effects and Mechanisms of MVO Through the Isolated Rat Thoracic Aorta Rings

#### 3.3.1 Preparation of Solution

Phenylephrine (PHE), acetylcholine chloride (Ach), methylene blue (MB), glibenclamide (Gli), propranolol, atropine sulfate, nifedipine, and N-nitro-L-arginine methyl ester (L-NAME) were prepared with ultrapure water. Indomethacin (Indo) and wortmannin were dissolved in 0.1% DMSO.

The Krebs–Ringer bicarbonate (K-H) solution (118.3 mM NaCl, 4.7 mM KCl, 2.5 mM CaCl_2_, 1.2 mM MgSO_4_, 1.2 mM KH_2_PO_4_, 25 mM NaHCO_3_, 0.5 mM EDTA, and 11.1 mM glucose) (Gao et al., 2007), a calcium-free K-H solution, was prepared by deleting CaCl_2_ from the K-H solution and replacing EDTA with EGTA. Notably, CaCl_2_ was added at the end after other dissolutions and the solvent was ultrapure water.

#### 3.3.2 Preparation of Isolated Thoracic Aorta Rings

Rats were anesthetized with 10% chloral hydrate and isolated the main thoracic aorta immediately, and the arteries were immersed in a cold oxygenated K-H solution. The arteries were cleared of connective tissues and cut into ring segments, 3–4 mm long. The rings were immersed in a temperature-controlled (37°C) chamber bath containing 20 ml K-H solution. During the course of the whole experiment, the solution was continuously oxygenated with a gas mixture of 95% O_2_ plus 5% CO_2_. The rings were equilibrated for 1 h at a resting tension of 1 g. During the equilibration period, the K-H solution was changed every 15 min. Then, the rings were contracted with 60 mM KCl and the process was repeated three times to stimulate the maximum contraction.

For de-endothelialized rings, the thoracic arteries were perfused with 0.1% Triton X-100 (0.1% Triton X-100 solution was obtained by dissolving 1ml Triton X-100 into 100 ml ultrapure water) for 10 s to remove the endothelium before being cutting into ring segments (Fountain et al., 2004; Wang et al., 2015). The integrity of the functional endothelium was tested by obtaining a relaxation to Ach (1 μM) in rings precontracted with PHE (10 μM). The endothelium was considered intact when such an Ach-induced relaxation was more than 85% of the pre-contraction value to PHE. In the experiments with de-endothelialized rings, the lack of relaxation or no more than 10% in response to Ach was considered to show the successful removal of the endothelium (Wang et al., 2015).

In all aortic ring experimental protocols, the concentrations represent the final tissue chamber concentration (containing 20 ml K-H solution).

#### 3.3.3 The Direct Effect of MVO on Resting Endothelium-Intact(+E) Rings

To observe the direct effect of MVO on the isolated rat aorta under normal conditions, an increasing concentration of MVO was added accumulatively (1.5, 2.25, 3, 3.75, and 4.5 μg/ml) to act directly on the +E rings in the MVO group after the initial equilibration of the suspended aortic rings. Similarly, an equal volume of DMSO was added and the highest cumulative concentration of DMSO was 0.015% (v/v) in the control group. Changes in the tension of aortic rings were recorded, and the relaxation rate was calculated.

#### 3.3.4 The Direct Effect of MVO on +E Rings or Endothelium-Denuded(-E) Rings

To determine the vasorelaxant effect of MVO, 60 mM KCl and 10 μM PHE was used to preconstrict stabilized +E or -E rings. After a sustained contraction was obtained, the concentration-dependent responses induced by MVO (1.5, 2.25, 3, 3.75, and 4.5 μg/ml) were examined. The same volume DMSO was applied, and the highest cumulative concentration of DMSO was 0.015% (v/v) in the control group. Changes in the tension of aortic rings were recorded, and the relaxation rate was calculated.

#### 3.3.5 Effect of MVO on + E Rings Pre-incubated By With Receptor-Related or Endothelium-Related Inhibitors

To examine the role of a β_2_-adrenoreceptor, muscarinic receptor, and the PI3K-Akt signaling in the vasorelaxant effect of MVO, +E rings were pretreated with 1 μM propranolol (β_2_-adrenoceptor antagonist), 0.1 μM atropine sulfate (a competitive nonselective muscarinic receptor antagonist), and 0.3 μM wortmannin (a PI3K-Akt inhibitor), respectively, for 20 min before the addition of PHE (10 μM). Then, MVO (1.5, 2.25, 3, 3.75, and 4.5 μg/ml) was added. Changes in the tension of aortic rings were recorded, and the relaxation rate was calculated. The vasorelaxant rates were compared with or without inhibitors.

To evaluate the role of the NO-related pathway, known concentrations of the antagonistic agents, involving 100 μM L-NAME (a nonspecific NOS inhibitor), 1 μM Indo (a nonselective cyclooxygenase inhibitor), and 10 μM MB (a cGMP inhibitor) were added to the tissue chamber 20 min before the addition of PHE (10 μM) and KCl(60 mM). Changes in the tension of aortic rings were recorded, and the relaxation rate was calculated. The vasorelaxant rates were compared with or without inhibitors.

#### 3.3.6 Effect of MVO on −E Rings Pre-incubated By Various Potassium Channel Blockers

To determine whether MVO-induced relaxation was correlated to the activation of K^+^ channels, four kinds of K^+^ channel blockers were selected to inhibit the different K^+^ channels, involving BaCl_2_(10^−3^M), TEA (10^−3^M), (Gli 10^−6^M), and 4-AP (10^−3^M), which were the blockers of K_IR_ (inward rectifier K^+^ channels), K_Ca_^2+^(calcium-activated K^+^ channels), K_ATP_ (ATP-sensitive K^+^ channels), and K_V_(voltage-dependent K^+^ channels), respectively. The -E rings were used in this experiment, pre-incubated with or without those four blockers for 20 min before the addition of PHE (10 μM). The accumulation concentrations of MVO (1.5, 2.25, 3, 3.75, and 4.5 μg/ml) were added after the vasocontraction of rings became stable. Changes in the tension of aortic rings were recorded, and the relaxation rate was calculated. The vasorelaxant rates were compared with or without blockers.

#### 3.3.7 Effect of MVO on Extracellular Ca^2+^-induced Contraction and Intracellular Ca^2+^ Release 

To determine whether the vasorelaxation of MVO was resulted from the regulation of Ca^2+^ releases, KCl(60 μM) and PHE(10 μM) were used to trigger the rings’ contraction after pre-incubation with MVO (4.5 μg/ml), nifedipine (4.5 μM), and DMSO(0.015% (v/v) for 20 min in Ca^2+^-free K-H solution. Changes in the tension of aortic rings were recorded, and the relaxation rate was calculated.

To test the effect of MVO on the regulation of Ca^2+^ influx, after pre-contraction with PHE (10 μM) and KCl (60 μM) and pre-incubation with MVO (4.5 μg/ml), nifedipine (4.5 μM), and DMSO(0.015% (v/v) for 20 min, cumulative concentrations of CaCl_2_(0.5–2.5 mM) were added in Ca^2+^ free K-H solution to trigger the contraction of -E rings. Changes in the tension of aortic rings were recorded, and the relaxation rate was calculated.

#### 3.3.8 Data Analysis and Statistics

The formula for calculating the degree of vasodilation was as follows: vasorelaxation rate (VR, %) = (Emax − Et)/Emax × 100%. Emax (the maximal relaxant magnitude) was equal to the maximum vasoconstriction force caused by vasoconstrictor. Et (the real-time relaxant magnitude) was equal to the vasoconstriction force at different concentrations of MVO. Data were presented as mean ± S.N. Student’s t-test (two-tailed) was used to compare the results in different groups. The difference comparison among multiple groups was tested by one-way ANOVA. A value of *p* < 0.05 was considered statistically significant.

## 4 Results

### 4.1 The Extraction Rate and Composition Analysis of MVO

**FIGURE 1:**
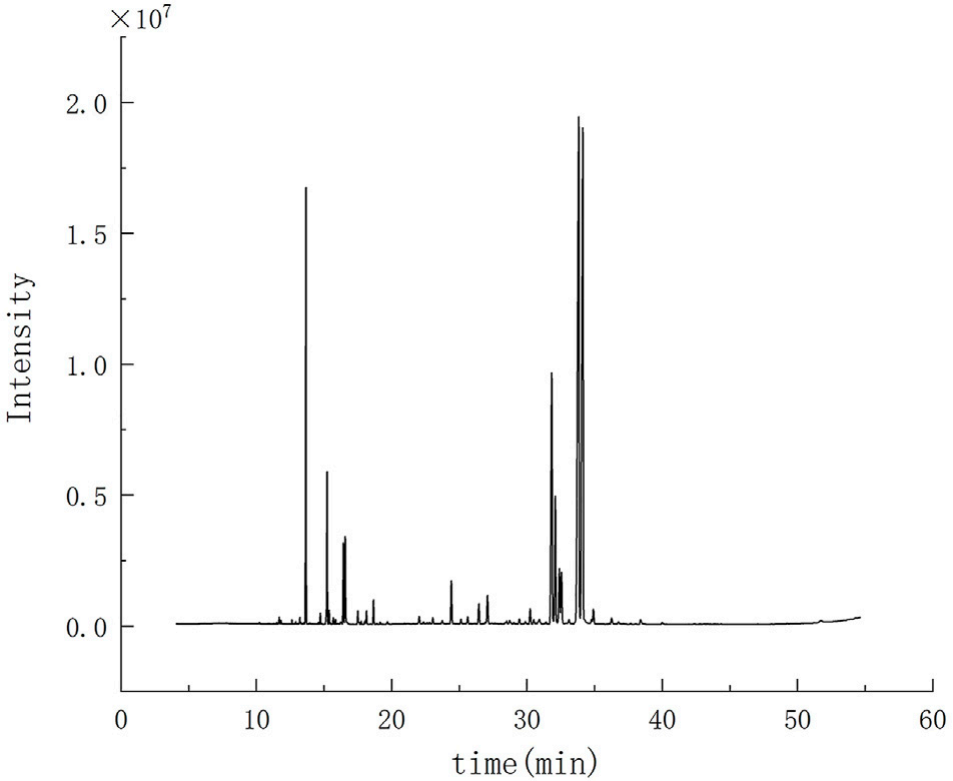
GC-MS chromatogram of MVO extracted from the bark of *M. officinalis* Rehd. et Wils.

There was about 1.55 ml MVO extracted from 500 g of the bark of *M. officinalis* Rehd. et Wils. According to the ER formulation, ER was 0.31%. The results of GC-MS were demonstrated in Figure 1. According to total iron chromatography, 29 components were determined, accounting for 95.46% of the total content, which was demonstrated in Table 1. Among them, there were 23 compounds with more than 0.1% and the oxygenated sesquiterpenes constituted the main portion of MVO, of which α-eudesmol, β-eudesmol, and γ-eudesmol were the top three ingredients in MVO with the highest content, accounting for 28.64%, 24.32%, and 10.53%, respectively.

**TABLE 1:** The chemical compositions in MVO extracted from the bark of *M. officinalis* Rehd. et Wils.

CAS number	Chemical name	Molecular formula	Remain time/min	The content of MVO/%
17,699-14-8	α-cubebene	C_15_H_24_	11.692	0.1
629-62-9	Pentadecane	C_15_H_32_	11.833	0.05
78-70-6	Linalool	C_10_H_18_O	12.633	0.07
118-65-0	Bicyclo [7.2.0]undec-4-ene,4,11,11-trimethyl-8-methylene-, (1R,4Z,9S)-	C_15_H_24_	13.21	0.14
87-44-5	Caryophyllene	C_15_H_24_	13.674	9.6
488-05-1	Elsholtzia ketone	C_10_H_14_O_2_	14.586	0.05
6753-98-6	α-humulene	C_15_H_24_	15.227	3.45
103,827-22-1	2-isoproenyl-4a,8-dimethyl-1,2,3,4,4a,5,6,7-octahydronaphthalene	C_15_H_24_	15.38	0.28
30,021-74-0	γ-muurolene	C_15_H_24_	15.686	0.14
17,066-67-0	(+)-β-selinene	C_15_H_24_	16.433	1.86
473-13-2	α-selinene	C_15_H_24_	16.568	2.08
483-76-1	(+)-δ-cadinene	C_15_H_24_	17.498	0.35
25,532-79-0	Trans-α-bisabolene	C_15_H_24_	17.992	0.09
2111-75-3	Perillyl aldehyde	C_10_H_14_O	18.139	0.35
73,209-42-4	(+/-)-trans-calamanene	C_15_H_22_	19.668	0.09
21,391-99-1	α-calacorene	C_15_H_20_	22.356	0.06
1139-30-6	Caryophyllene oxide	C_15_H_24_O	24.415	1.43
19,888-34-7	(1R,3Z,7Z,11S)-1,5,5,8-tetramethyl-12-oxabicyclo [9.1.0]dodeca-3,7 -diene	C_15_H_24_O	26.45	0.71
913,176-41-7	Caryophyllenyl alcohol	C_15_H_26_O	27.068	1.01
77-53-2	Cedrol	C_15_H_26_O	29.438	0.2
1209-71-8	γ-eudesmol	C_15_H_26_O	31.832	10.53
17,334-55-3	(+)-calarene	C_15_H_24_	32.091	4.84
31,983-22-9	α.-muurolene	C_15_H_24_	32.379	2.21
23,811-08-7	Hinesol	C_15_H_26_O	32.55	1.96
473-16-5	α-eudesmol	C_15_H_26_O	33.65	28.64
473-15-4	β-eudesmol	C_15_H_26_O	34.138	24.32
5945-72-2	(-)-selin-11-en-4alpha-ol	C_15_H_26_O	34.908	0.58
19,431-79-9	5α-hydroxycaryophylla-4(12),8(13)-diene	C_15_H_24_O	36.702	0.14
57-10-3	Palmitic acid	C_16_H_32_O_2_	51.754	0.13

### 4.2 The Results of Network Pharmacology Predictions

#### 4.2.1 Targets of the Active Ingredients of MVO

A total of 23 components of MVO were identified and selected. However, there were 3 ingredients deleted because perillyl aldehyde and (-)-selin-11-en-4alpha-ol as well as the target of γ-eudesmol could not be found in the TCMSP database, and 20 other ingredients were selected as the active compounds of MVO for further analysis. Moreover, after the duplicate values were removed, 37 targets of active compounds of MVO were obtained from the TCMSP database and transformed into the gene names *via* the Uniprot database. The detailed information of the active compounds of MVO was listed in Supplementary Table S1.

#### 4.2.2 The Construction and Analysis of the C-T Network

**FIGURE 2:**
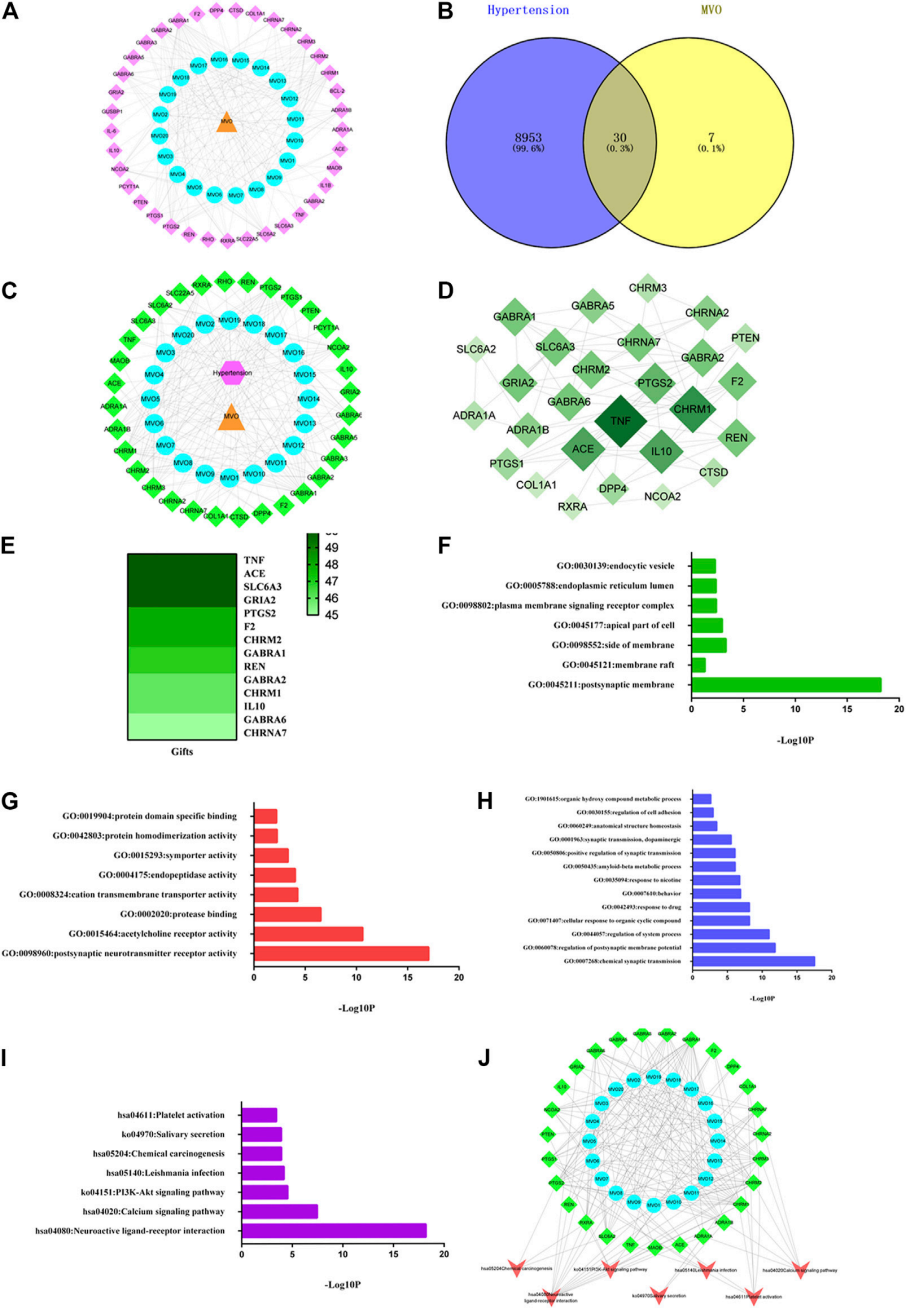
MVO potential target–hypertension target network and analysis. **(A)** The C-T network of MVO. **(B)** The Venn diagram. **(C)** The C-T-D network of MVO. **(D)** The PPI network of the 30 overlapping targets. **(E)** The heatmap of gifts between hypertension and hub target. **(F)** The GO-CC enrichment analysis. **(G)** The GO-MF enrichment analysis. **(H)** The GO-BP enrichment analysis. **(I)** The KEGG enrichment analysis. **(J)** The C-T-P network of MVO. The orange triangle node represented MVO, the blue circle node represented each compound of MVO, the pink diamond node represented the targets of MVO, the pink hexagon refers to hypertension, and the green diamond node represented the overlapping targets of MVO and hypertension; the red V was the symbol of pathways.

The C-T network was built by mapping 20 active compounds to their 37 corresponding potential targets to get a systematic and holistic interaction between the active compounds of MVO and their potential targets. As shown in Figure 2A, the network consisted of 58 nodes (1 herbal medicine node, 20 main compound nodes, and 37 compound-associated target nodes) and 190 interaction edges. In the network, the degree of eight compounds was more than 10. In detail, beta-caryophyllene (degree 17), palmitic acid, and (+)-β-selinene (degree 14), bicyclo [7.2.0]undec-4-ene,4,11,11-trimethyl-8-methylene-, (1R,4Z,9S)- (degree 13), maryophyllene oxide, α-muurolene, α-selinene, and 2-isopropenyl-4a,8-dimethyl-1,2,3,4,4a,5,6,7-octahydronaphthalene (degree 11) presented the maximum interactions with potential targets, revealing that these compounds with high degree values might exert an important role in the potential pharmacological effects of MVO. Moreover, five targets with degrees more than 10 were found, including GABRA1(degree 19), CHRM1 (degree 14), PTGS2 (degree 13), CHRM2 (degree 12), and CHRM3 (degree 11). Each target was connected with a variety of compounds. The C-T network revealed intimate communications between those compounds and their related targets, providing a reference to further investigate the pharmacological mechanisms of MVO.

#### 4.2.3 The Construction and Analysis of the C-T-D Network

A total of 8,983 targets associated with hypertension were identified from the GeneCards, OMIM, and TTD databases after duplicate targets were deleted (Supplementary Table S1). And then, the Venn graph was built, as pictured in Figure 2B. Furthermore, according to the 30 overlapping targets of MVO and hypertension, the C-T-D network was constructed, which consisted of 54 nodes (1 hypertension node and one herbal medicine note and 52 overlapping target nodes) and 214 edges. As shown in Figure 2C, the top nine targets with the degree values ≥10 were regarded as the most pivotal targets in the C-T-D network, namely, GABRA1, CHRM1, PTGS2, CHRM2 CHRM3, SLC6A2, GABRA6, NCOA2, and GABRA2. Thereby, the above targets might be the potential targets for MVO to treat hypertension.

#### 4.2.4 The Construction and Analysis of PPI Network

The PPI network was constructed to better interpret the mechanisms of MVO in hypertension treatment by using STRING software, as shown in Figure 2B. Thirty overlapping targets were used to establish a PPI network after importing into the STRING database. As shown in Figure 2D, the PPI network consisted of 27 nodes and 70 edges. Based on the degree principle of each target, TNF, CHRM1, ACE, IL10, PTGS2, REN, F2, SLC6A3, GABRA6, GRIA2, CHRNA7, GABRA1, CHRM2, and GABRA2 were determined as the hub’s top 14 targets. Moreover, we assessed the associations between hub targets and hypertension by the Genecards database. As it was shown in Figure 2E, these hub targets were closely corresponded with hypertension according to gifts.

#### 4.2.5 The Analysis of Gene Ontology Enrichment and Construction of C-T-P Network

To explore the functional role of the 30 overlapping targets, GO functional analysis were conducted *via* the Metascape database. The results of GO enrichment analysis involved 48 cell components (CCs), 50 molecular functions (MFs), and 286 biological processes (BPs) with a threshold value of *p* < 0.05. The top GO analysis results are shown in Figures 2F–H. The CC analysis demonstrated that the overlapping targets were mainly related to the postsynaptic membrane, membrane raft, side of the membrane, apical part of a cell, plasma membrane signaling receptor complex, endoplasmic reticulum lumen, and endocytic vesicle. The MF results mainly included postsynaptic neurotransmitter receptor activity, acetylcholine receptor activity, protease binding, cation transmembrane transporter activity, endopeptidase activity, symporter activity, protein homodimerization activity, and protein domain specific binding. The BP results mainly comprised of chemical synaptic transmission, the regulation of postsynaptic membrane potential, regulation of system process, cellular response to organic cyclic compound, response to drug, behavior, response to nicotine, amyloid-beta metabolic process, positive regulation of synaptic transmission, synaptic transmission, dopaminergic, anatomical structure homeostasis, regulation of cell adhesion, and organic hydroxy compound metabolic process.

The results of the KEGG enrichment analysis revealed that seven pathways met the threshold value of *p* < 0.05 after sorting according to the *P*-value. As depicted in Figure 2I, the KEGG pathways of MVO against hypertension were mainly related to neuroactive ligand–receptor interaction, the calcium signaling pathway, PI3K-Akt signaling pathway, Leishmania infection, chemical carcinogenesis, salivary secretion, and platelet activation.

The results of the C-T-P network were demonstrated in Figure 2J. There were 54 nodes and 199 edges in the C-T-P network, including 20 active compounds of MVO nodes, 27 overlapping target notes, and seven pathway nodes. The top three degrees were neuroactive ligand–receptor interaction (degree = 13), the calcium signaling pathway and platelet activation (degree = 6), and PI3K-Akt signaling pathway (degree = 5), respectively, which might be mainly responsible for the vasorelaxant effect of MVO.

### 4.3 The Results of Vasorelaxation Effects and Mechanisms of MVO

#### 4.3.1 Effects of MVO on Thoracic Aortic Rings With 1 g Resting Tension

**FIGURE 3:**
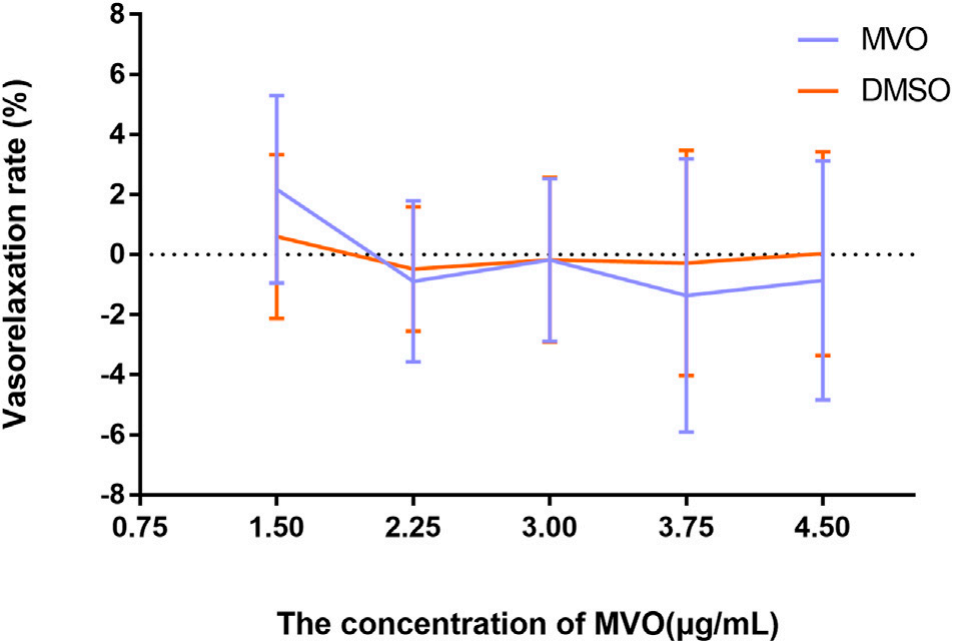
Effects of MVO (1.5, 2.25, 3, 3.75, and 4.5 μg/ml) on thoracic aortic rings with 1 g resting tension. *n* = 6. Means ± S.D. *p < 0.05, **p < 0.01 vs. DMSO group.

The accumulative addition of MVO (1.5, 2.25, 3, 3.75, and 4.5 μg/ml) had no significant effects on rat thoracic aortic rings-sustained basal tension when compared to the vehicle control group (*p* > 0.05) (Figure 3).

#### 4.3.2 Effects of MVO on +E Rings Pre-contracted By KCl and PHE

**FIGURE 4:**
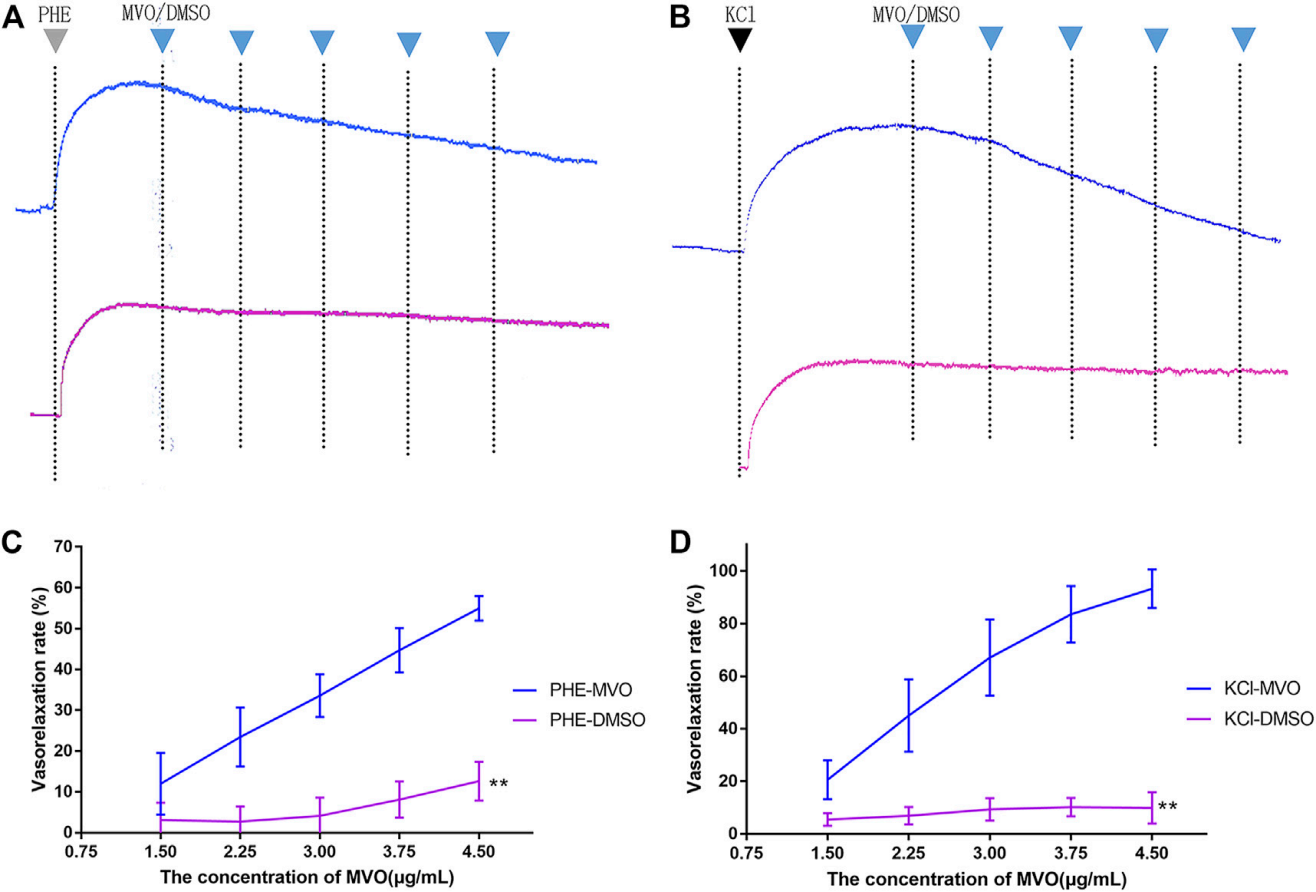
Effect of cumulative doses of MVO (the blue line) and DMSO (the purple line) on the relaxation of rat aortas precontracted by **(A)** 10 μM PHE and **(B)** 60 mM KCl. **(C)** Relaxant effects of MVO (1.5, 2.25, 3, 3.75, and 4.5 μg/ml) on contractions induced by PHE (10 μM). The alternation of tension was expressed as the percentage of the active contractions induced by PHE (10 μM). **(D)** Relaxant effects of MVO (1.5, 2.25, 3, 3.75, and 4.5 μg/ml) on contractions induced by KCl (60 mM). The alternation of tension was expressed as a percentage of the active contractions induced by KCl (60 mM). *n* = 6. Means ± S.D. *p < 0.05, **p < 0.01 vs. KCl/PHE-MVO group.

In Figure 4, MVO produced relaxation in +E rings in a concentration-dependent manner pre-contracted with PHE (Figure 4A) and KCl (Figure 4B). When the rings contracted with PHE (10 μM), the Emax for MVO was 54.97% ± 3.03%, and the PHE-DMSO was 15.15% ± 3.66% (Figure 4C). Additionally, when the rings contracted with KCl (60 mM), the Emax for MVO was 91.96% ± 5.38%, while the Emax for DMSO was 9.93% ± 5.92% (Figure 4D). In conclusion, the statistical differences were shown in arterial rings with the control group compared with the treatment of KCl/PHE-MVO groups (**p < 0.01), revealing that MVO elicited concentration-dependent relaxations in the arterial segments precontracted by KCl or PHE under +E conditions.

#### 4.3.3 Vasorelaxant Effect of MVO on -E and +E Rings Pre-contracted By KCl and PHE

**FIGURE 5:**
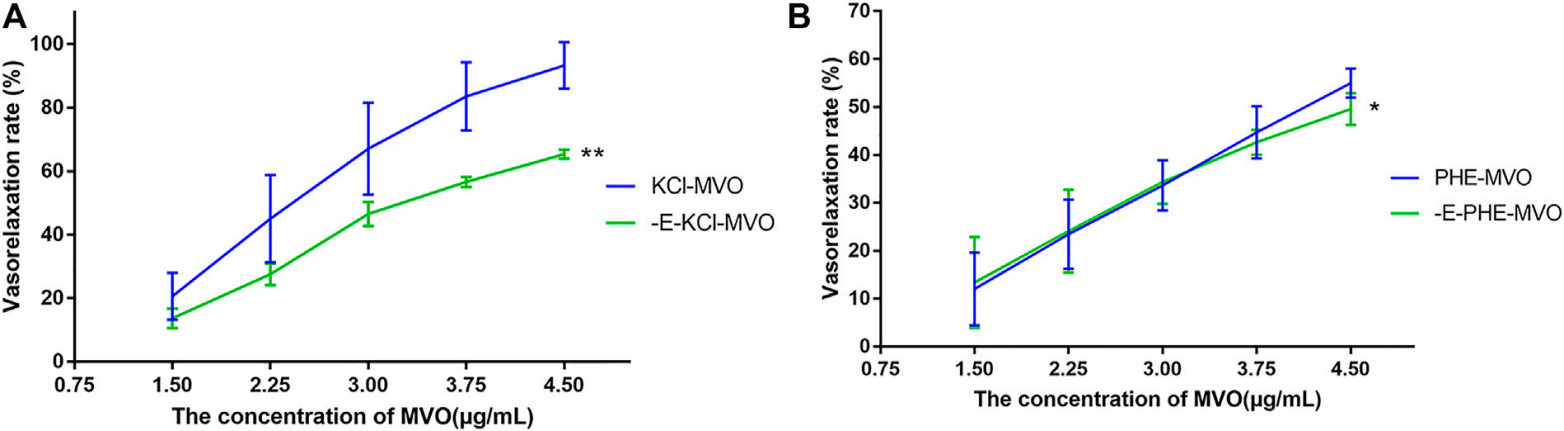
Relaxant effects of MVO on +E or -E aortic rings precontracted with **(A)** KCl (60 mM) or **(B)** PHE (10 μM). The alternation of tension was expressed as a percentage of the active contraction induced by KCl (60 mM) or PHE (10 μM). *n* = 6. Means ± S.D. *p < 0.05, **p < 0.01 vs. KCl/PHE-MVO group.

Compared with VR in rings with endothelium, the vasorelaxant effects of MVO obviously decreased and were statistically significant in -E arterial rings (Figure 5). The VR of -E-KCl-MVO decreased by about 25% (Emax/KCl: +E, 91.96% ± 5.38% vs. -E, 65.74% ± 3.27%, *p* < 0.01), and the VR of -E-PHE-MVO decreased by about 5% (Emax/PHE: +E, 54.97% ± 3.03% vs. -E, 49.58% ± 3.31%, *p* < 0.05).

#### 4.3.4 Vasorelaxant Effect of MVO on +E Rings Pre-incubated By Receptor-Related Inhibitors

**FIGURE 6:**
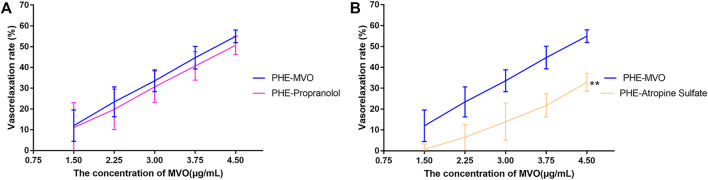
Effects of **(A)** propranolol (1 μM) or **(B)** atropine sulfate (1 μM) on MVO-produced relaxant effects in +E aortic rings precontracted with PHE (10 μM). The alternation of tension was expressed as the percentage of the active contraction induced by PHE (10 μM). *n* = 6. Means ± S.D. *p < 0.05, **p < 0.01 vs. PHE-MVO group.

Pre-incubation with propranolol (1 μM) did not significantly regulate MVO-produced vasorelaxation (Emax: 50.65% ± 4.43% vs. 54.97% ± 3.03% in the PHE-MVO group, *p* > 0.05, which was depicted in Figure 6A, suggesting minimal contribution by β-adrenoceptors to the MVO-induced vasorelaxation.

Pre-incubation with atropine sulfate (1 μM), a muscarinic receptor antagonist, did downregulate MVO-produced vasorelaxation (Emax: 32.87% ± 4.33% vs. 54.97% ± 3.03%, *p* < 0.01 (Figure 6B), which proved that the M receptor was responsible for the vasorelaxant effect of MVO.

#### 4.3.5 Vasorelaxant Effect of MVO on +E Rings Pre-incubated By Endothelium-Related Inhibitors

**FIGURE 7:**
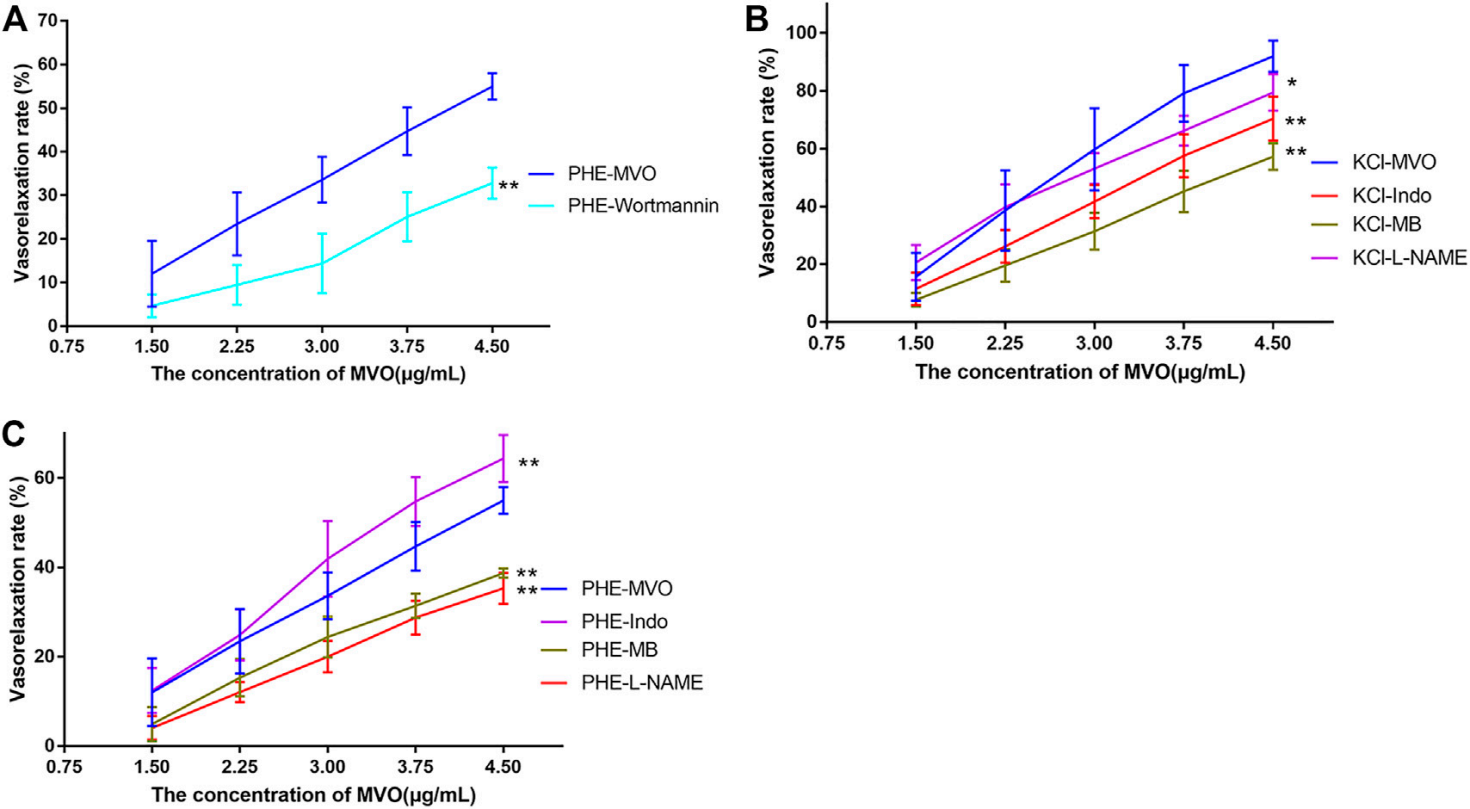
Effects of MVO on rings pre-incubated by different inhibitors. **(A)** 0.3 μM wortmannin (a PI3K/Akt inhibitor) precontracted with PHE, 100 μM L-NAME (an NOS inhibitor), 1 μM Indo (a nonselective COX inhibitor), and 10 μM MB (a COX inhibitor) on MVO-produced relaxatory effects in +E aortic rings pre-contracted by **(B)** KCl (60 mM) or **(C)** PHE (10 μM). The alternation of tension was expressed as a percentage of the active contraction induced by KCl (60 mM) or PHE (10 μM). *n* = 6. Means ± S.D. *p < 0.05, **p < 0.01 vs. KCl/PHE-MVO group.

Pre-incubation with wortmannin (0.3 μM) did significantly downregulate MVO-produced vasorelaxation (Emax: 54.97% ± 3.03% vs. 32.8% ± 3.57%, *p* < 0.01 (Figure 7A), which demonstrated that PI3K-Akt might play a vital role in the MVO vasorelaxant effect.

As displayed in Figure 7B, the pre-incubation of +E arterial rings with Indo, L-NAME, or MB did inhibit MVO-produced relaxation (Emax/KCl-Indo: +E, 70.35% ± 7.59%, *p* < 0.05; E_max_/KCl-MB: +E, 57.26% ± 4.61%, *p* < 0.05; Emax/KCl-L-NAME: +E, 79.48% ± 6.35%, *p* < 0.05) vs. the KCl-MVO group. Similarly, pre-contraction with PHE and pre-incubation with L-NAME or MB did inhibit MVO-produced relaxation (Emax/PHE-L-NAME: +E, 36.51 ± 3.49%; Emax/PHE-MB: +E, 38.72 ± 1.03%; *p* < 0.01). On the contrary, Indo did accelerate MVO-induced relaxation precontracted by PHE (Emax/PHE-Indo: +E, 64.37 ± 5.23%, *p* < 0.01) vs. the PHE-MVO group shown in Figure 7C.

#### 4.3.6 Vasorelaxant Effect of MVO on −E Rings Pre-incubated By Various Potassium Channel Blockers

**FIGURE 8:**
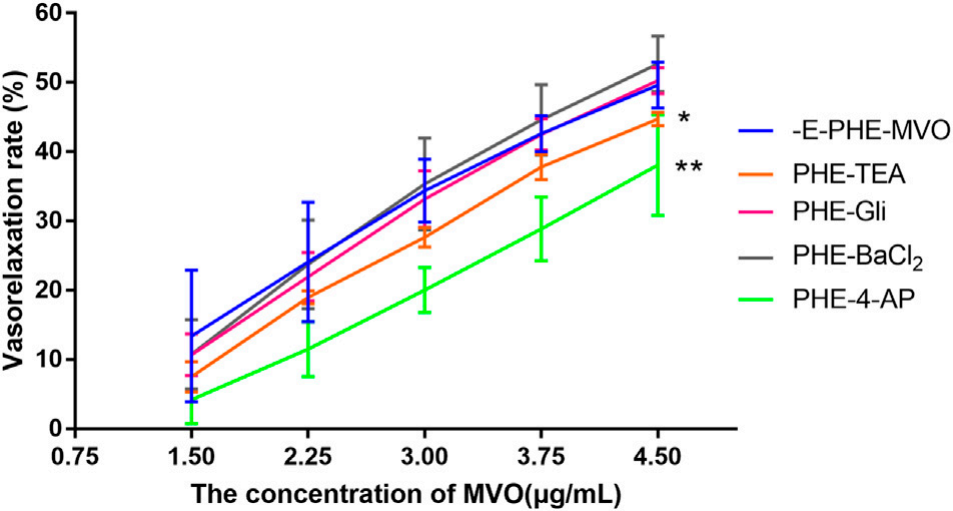
Influences of different K^+^ channel inhibitors, including Ca^2+^-activated K^+^ (K_Ca_) channel inhibitor TEA (10 mM), voltage-dependent K^+^ (K_V_) channel inhibitor 4-AP (1 mM), ATP-sensitive K^+^ (K_ATP_) channel inhibitor glyburide (10 μM), and inward rectifier K^+^ (K_IR_) channel inhibitor BaCl_2_ (100 μM) on MVO-produced relaxant effects in -E aortic rings precontracted with PHE (10 μM). The alternation of tension was expressed as the percentage of the active contraction induced by PHE (1 μM). *n* = 6. Means ± S.D. *p < 0.05, **p < 0.01 vs. -E-PHE-MVO group.

After pre-incubation with TEA (10 mM), Gli (10 μM), or BaCl_2_ (100 μM) for 20 min, the Emax values that MVO produced were 44.71% ± 0.99%, 53.45% ± 6.26%, and 52.69% ± 4.01%, respectively, which had no statistical significance as compared to the -E-PHE-MVO group (Emax 49.58 ± 3.31%, *p* > 0.05), suggesting that K_Ca_, K_ATP_, and K_IR_ did not participate in the vasorelaxant effect of MVO. In contrast, after the pre-incubation of 4-AP (1 mM) for 20 min, the E_max_ values that MVO produced were 37.31 ± 6.16%, which had a statistical significance as compared to the -E-PHE-MVO group (Emax 49.58 ± 3.31%, *p* < 0.05) (Figure 8), showing that the activation of Kv channel participated in the vasorelaxant effect of MVO in this study.

#### 4.3.7 Effect of MVO on −E Rings in Ca^2+^-free K-H Solution

**FIGURE 9:**
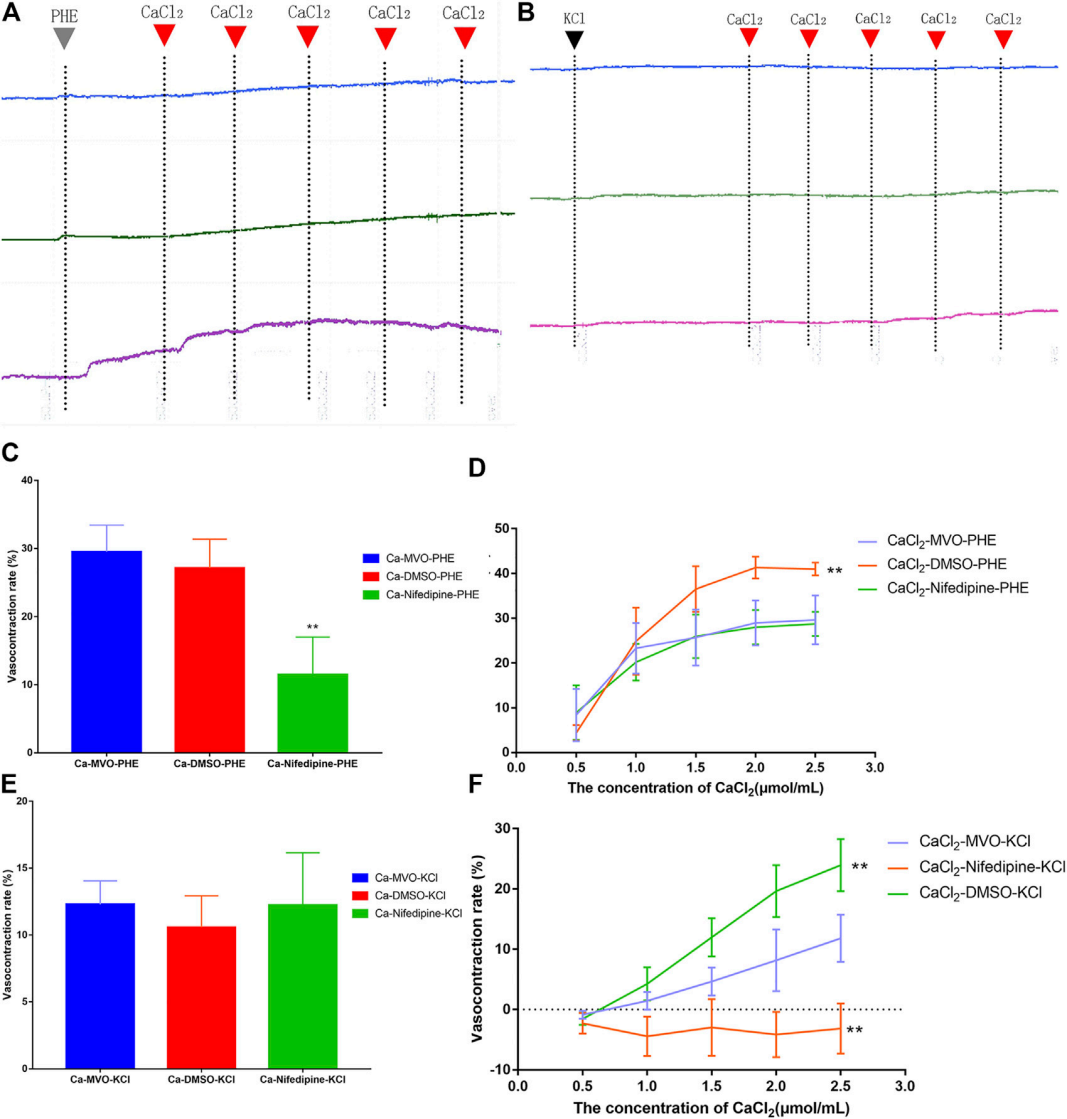
The effect of calcium signaling pathway on vasorelaxation effect of MVO (4.5 μg/ml, the blue line), nifedipine (4.5 μM, the green line), and DMSO (0.015% (v/v), the purple line) on the **(A)** PHE (10 μM) or **(B)** KCl (60 mM)-induced contractions of -E aortic rings in Ca^2+^-free solution. Inhibitory effects of MVO (4.5 μg/ml) on the **(C)** PHE (10 μM) or **€** KCl (60 mM)-induced contractions of -E aortic rings in Ca^2+^-free solution. *n* = 6. Means ± S.D. *p < 0.05, **p < 0.01 vs. Ca-MVO-PHE/KCl group. Inhibitory effects of MVO (4.5 μg/ml) on the CaCl_2_ (0.5–2.5 mM)-induced contractions of -E aortic rings depolarized with **(D)** PHE (10 μM) or **(F)** KCl (60 mM) in Ca^2+^-free solution. *n* = 6. Means ± S.D. *p < 0.05, **p < 0.01 vs. CaCl_2_-MVO-PHE/KCl group.

In a Ca^2+^-free solution, there was no significant difference between MVO and the DMSO group in the short-term contraction caused by PHE (Figures 9A,C) or KCl (Figures 9B,E) (*p* > 0.05). Compared with the Ca-Nifedipine-PHE group, there was a significant difference in the Ca-MVO-PHE group (*p* < 0.01). The above results suggested that MVO relaxed aortic rings without releasing calcium from the sarcoplasmic reticulum.

In a Ca^2+^-free solution, the accumulative addition of CaCl_2_ induced concentration-dependent contractions of -E aortic rings depolarized with 10 μM PHE or 60 mM KCl. Pretreatment with 4.5 μg/ml MVO and nifedipine could significantly antagonize Ca^2+^-induced contractions, which was shown in Figures 9A,B. Among them, there was a statistical difference between the CaCl_2_-MVO-PHE group (29.63 ± 5.45%) and the CaCl_2_-DMSO-PHE group (40.98 ± 1.47%), *p* < 0.01) (Figure 9D). In addition, the CaCl_2_-MVO-KCl group (11.82 ± 3.92%) showed a significant difference from the CaCl_2_-DMSO-KCl group (23.95 ± 4.34%) and the CaCl_2_-nifedipine-KCl group (-3.14 ± 4.16%), (*p* < 0.01) (Figure 9F). Therefore, these results showed that MVO exerted a vasorelaxant effect by inhibiting external calcium influx to a certain extent.

## 5 Discussion

The present study explored the vasorelaxant activity of MVO in rat aorta. The compounds of MVO were initially analyzed by GC-MS technology, and network pharmacology analysis was applied to predict the active ingredients, the targets, the biological processes, and the pathways of MVO on hypertension. The perfusion experiment of the isolated thoracic aortic ring was selected to verify the predictions of network pharmacology.

There may be quality differences between Chinese herbs from different habitats. Thus, to avoid the influence of the quality of the tested medicinal materials on the experimental results, the dry rind of *M. officinalis* Rehd. et Wils. from seven different main producing areas were mixed in equal proportions as experimental medicinal materials. According to GC-MS results, the composition of MVO was consistent with that in other studies. For example, our study demonstrated that α-, β-, and γ-eudesmol were the main ingredients of MVO, and the ingredients with considerably higher contents were mainly β-caryophyllene, (+)-calarene, and α-humulene (Yang et al., 2007; Xu et al., 2009; Lou et al., 2011; Fang et al., 2012; He et al., 2012; Tang et al., 2019), suggesting that the quality of MVO in our study was representative and reliable. Previous studies have found that β-caryophyllene is a type of bicyclic sesquiterpenoids that has good application value in cardiovascular and cerebrovascular diseases by eliciting anti-inflammatory (Basha and Sankaranarayanan, 2016) and neuroprotective effects (Zhang et al., 2017; Machado et al., 2018), which are directly and indirectly related to vascular disease and hypertension (Ohbuchi et al., 2015; Scholl et al., 2015; Jiye Chen et al., 2021). Then, those ingredients in MVO were used to explore its vasorelaxant activity and mechanisms *via* network pharmacology. For one thing, although α-, β-, and γ-eudesmol had the highest content of MVO, they were not the active ingredients predicted by network pharmacology. For another thing, although α-caryophyllene was regarded as one of the most possible ingredients to vasorelaxant vessels proved by network pharmacology, its vasorelaxant effect has not been confirmed. Moreover, the results showed that the same target was connected to multiple ingredients in MVO, suggesting that the active ingredients in MVO were likely to have an interaction with the treatment of hypertension. Therefore, MVO, rather than beta-caryophyllene or other monomer compounds, was used to do experiments *in vitro*.

In the PPI network, a total of 14 targets, such as TNF, CHRM1, ACE, IL10, PTGS2, REN, and F2, were the hub targets of MVO for treating hypertension. Studies have shown that those targets are closely related to thrombus formation and inflammation, which are involved in the development of hypertension (Otterdal et al., 2008). Long-term inflammation can increase the production of ROS, cause oxidative stress and endothelial dysfunction, (Zhang et al., 2018) and easily result in hypertension (Fujita, 2014; Gomolak and Didion, 2014). Furthermore, the inflammation is characterized by increased levels of local inflammatory cytokines such as TNF (Davis et al., 2002), IL10 (Di et al., 2020), and PTGS2 (Simon, 1999), which are more likely to be useful diagnostic tools for hypertension in the future. Above all, these results provide preliminary evidence for illuminating the multi-targeted mechanisms of the anti-hypertensive effect of MVO.

In the KEGG pathway analysis, overlapping targets’ enrichment in hypertension-associated pathways mainly included neuroactive ligand–receptor interaction, the PI3K-Akt signaling pathway, and calcium signaling pathway, which played a vital role in the management of hypertension. Substantial evidence has shown that the treatment of hypertension was closely regulated by the activation of the Src-PI3K/Akt-eNOS signaling pathway (Hao et al., 2017) and blockage of the Ca^2+^ channel (Zhou et al., 2019), which were consistent with the prediction of network pharmacology. Although network pharmacology analysis is a widely used and acceptable method to predict the therapeutic mechanism, it is necessary to confirm the correctness of the prediction due to the possible different standards and spurious associations between one database and another (Luo et al., 2020). The first step was to verify whether MVO had a vasodilator function and whether this function was related to the function of these predicted targets and pathways. Thus, it is vital for us to carry out *in vitro* vasodilatory experiments.

However, the drawback of the investigation for the mechanism underlying the MVO-induced vasorelaxation was its heavy dependence on the pharmacological inhibitors. Therefore, it was important to make some prediction *via* network pharmacology before the mechanism research. Fortunately, the previous network pharmacology in our study provided directions and clues for the mechanism research, making it more targeted. However, it was worth noting that DMSO has vasodilatory activity at high concentrations (0.1%–3%) (Kaneda et al., 2016). Therefore, as a solvent and control group, it is important to control the concentration of DMSO. Wang et al. (2015) wrote that “The time-matched vehicle control (DMSO) group was also analysed. The final concentration of DMSO was 0.1% (v/v)” in their study. However, DMSO (0.015% v/v) was the highest cumulative concentration of the control group in our study, suggesting that the vasorelaxant activity of the tested solution in the experiment was mainly due to the vasorelaxant effect of MVO instead of DSMO. We firstly evaluated the vasorelaxant effect of MVO in rat thoracic aorta (Zhong Yan Zhou et al., 2019). As was illustrated in Figure 4, MVO relaxed KCl- and PHE-induced vasocontraction and the vasorelaxation effect was more effective in KCl-contracted rat aorta, which preliminarily showed that MVO exactly exerted a vasorelaxant effect. Secondly, in order to verify the pathways of KEGG, atropine sulfate, a nonselective muscarinic receptor antagonist, was used to verify neuroactive ligand–receptor interaction. Neuronal excitability is directly linked to the ion channel state of the central nervous system, which is closely related to neuronal signaling and the regulation of blood pressure (Scholl et al., 2015). The results showed that there was a significant difference in the VR (%) between the two groups with or without atropine sulfate, which was shown in Figure 6B, revealing that MVO exactly exerted vasorelaxant effects through the activating muscarinic receptors. However, the specific receptor subtype was not clear because atropine sulfate is a competitive nonselective muscarinic receptor antagonist, and the muscarinic limb of the parasympathetic system not only contracts smooth muscle directly *via* activation of M3 receptors (Murthy and Makhlouf, 1997) but also abrogates sympathetically mediated relaxation *via* the activation of M2 receptors (Kume and Kotlikoff, 1991). Thirdly, wortmannin and several endothelium-related inhibitors (involving Indo, MB, and L-NAME) were selected to study the effect of the PI3K-Akt-NO pathway. The motivation of the PI3K-Akt pathway is associated with the phosphorylation of eNOS that causes the production and release of NO, vascular integrity, as well as regulation of blood pressure homeostasis (Peixoto et al., 2017; Lee et al., 2018). Our experiment showed the VR (%) was significantly different in the presence or absence of wortmannin, an inhibitor of the PI3K-Akt signaling involved in the vasorelaxant effect (Hao et al., 2017), suggesting that the activation of the PI3K-Akt pathway was of the MVO to relax aortic rings. Additionally, L-NAME (a nonspecific NOS inhibitor) and MB (a cGMP inhibitor) partly inhibited the vasorelaxation of MVO precontracted by KCl or PHE, which indicated that MVO exerted a vasorelaxant effect through promoting NO release and cGMP activity in part. Notably, Indo (a nonselective cyclooxygenase inhibitor) showed a completely different effect precontracted with PHE and KCl. Compared with the control group, the VR (%) in Indo-PHE was higher than that of the PHE-MVO group, while Indo-KCl was lower than that of KCl-MVO, which might be correlated with their different vasorelaxant mechanisms. In detail, KCl contracted rat rings through depolarizing the membrane, and the opening of the voltage-dependent Ca^2+^channels (VDCCs) (Eichhorn and Dobrev, 2007) while PHE acted on α1-adrenoceptors and subsequently motivated receptor-operated Ca^2+^channels (ROCCs) (Lee et al., 2010). In conclusion, the PI3K-Akt-NO pathway might be responsible for the vasorelaxant effect of MVO, which was consistent with the results of KEGG. Lastly, a Ca^2+^-free solution was applied to study whether the activation of calcium ion channels was involved in the vasorelaxant effect of MVO. The constriction of vascular smooth muscle is initiated by an increase of the concentration of Ca^2+^([Ca^2+^]i), and the calcium signaling pathway is expressed in virtually all vascular smooth muscle cells (VSMCs), and they are primarily activated by an elevation of [Ca^2+^]i due to the change of membrane potential (Pérez et al., 2001; Quilley and Qiu, 2005) or agonist-stimulated (Ganitkevich and Isenberg, 1990) Ca^2+^ release from intracellular stores. Importantly, calcium ion channels are divided into VDCCs and ROCCs, which can be activated by KCl and PHE, respectively (Anfinogenova et al., 2004; Thorneloe and Nelson, 2005; Lee et al., 2010). To determine whether the vasorelaxant activity of MVO resulted from the regulation of Ca^2+^, the effect of MVO on the contractile response induced by PHE and KCl and the addition of CaCl_2_ in Ca^2+^-free solution was studied. As shown in the results, compared with the control group, incubation with MVO caused an almost negligible effect shift of VR (%) in Ca^2+^-free solution precontracted by PHE and KCl, suggesting that the release of Ca^2+^ from the sarcoplasmic reticulum played a negligible role in the vasorelaxant activity of MVO. Differently, MVO exerted its vasorelaxant effect *via* inhibiting the external Ca^2+^ influx.

Moreover, the removal of endothelium partially attenuated MVO-induced relaxation, suggesting that VSMC-mediated vasodilation mechanisms, including the β_2_-adrenoreceptor, potassium ion channels, and calcium ion channels (Tang et al., 2021), were likely involved in the vasorelaxant activity of MVO. Therefore, in addition to calcium ion channels, which were predicted by the results of network pharmacology, the effect of β_2_-adrenoreceptor and potassium ion channels was explored *via* the application of different blockers. For one thing, β-adrenoceptor agonists elicited vasodilatory activity *via* activating adenylyl cyclase and consequently increasing cAMP in VSMCs (Huang and Kwok, 1998). As shown in Figure 6A, the β_2_-adrenoreceptor played no role in the vasorelaxant effect of MVO, which was confirmed by the usage of propranolol. For another thing, there are four kinds of the K^+^ pathway, involving K_IR_ (inward rectifier K^+^ channels), K_Ca_ (calcium-activated K^+^ channels), K_ATP_ (ATP-sensitive K^+^ channels), and K_V_ (voltage-dependent K^+^ channels), which can be blocked by BaCl_2_, TEA, Gli, and 4-AP, respectively. Among them, Kv exerts a dominant effect on determining the resting membrane potential and basal regulation on certain VSMCs (Park et al., 2010). In this study, the vasorelaxant effect of MVO was significantly inhibited by 4-AP, a K_V_ channel blocker, However, K_ATP_, K_Ca_, and K_IR_ played no role in the vasorelaxant effect of MVO, which was confirmed by the usage of Gli, TEA, and BaCl_2_, respectively.

**FIGURE 10:**
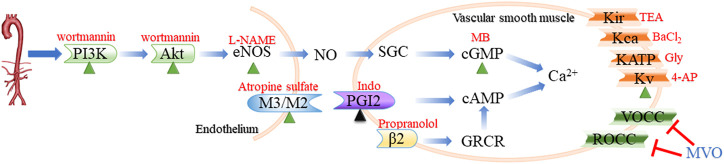
Routes of vasodilation mechanisms that participated in the vasorelaxation of MVO. The green triangle indicated that the activation of the mechanism was conducive to MVO’s vasorelaxant effect, while the black triangle suggested whether the activation of the mechanism was beneficial to MVO’s vasorelaxant effect depending on the type of pre-constrictor. The red T indicated that MVO exerted a vasorelaxant effect by inhibiting this mechanism. Blue words denoted MVO, and red words denoted common blockers of the corresponding pathways. eNOs, endothelial nitricoxide synthase; SGC, soluble guanylyl cyclase; cGMP, cyclic 3′,5′-guanosine monophosphate; PGI2, prostaglandin 2; cAMP, cyclic adenosine 3′, 5′-monophosphate; L-NAME, nitro-L-arginine; MB, methylene blue; Indo, indomethacin; Gly, glibenclamide; 4-AP, 4-aminopyridine; TEA, tetraethylammonium.

Collectively, our integrative approach elucidated the potential mechanisms of MVO against hypertension based on a systematic network perspective, and the novel findings regarding an acute vasorelaxant effect of MVO were demonstrated (Figure 10). According to the results of network pharmacology and experiment *in vitro*, MVO induced the relaxation of arterial rings in a dose-dependent manner endothelium dependently and independently, which were identified by the following four observations: 1) PI3K-Akt-NO-related and muscarinic receptor-related mechanisms were responsible for MVO-induced vasorelaxation partially; 2) the K_V_ channel participated in the vasorelaxation of MVO; 3) MVO-suppressed Ca^2+^ influx in rat mesenteric arteries rings contributed to the vasorelaxation effects of MVO; and 4) the release of Ca^2+^ or β_2_-adrenoceptors had little effect on the vasorelaxation of MVO. Our results firstly revealed that the underlying mechanisms of MVO-induced vasorelaxation were multiple for the first time *via* the combined application of network pharmacology and experiment, which directly pointed out the research direction and obtained reliable results (Wang et al., 2019; Jiang et al., 2021). Indeed, as previously noted, the two types of investigation have limitations. Therefore, a combination of the two types should be applied so that both of them can make up for each other’s shortcomings. Actually, the combined approach found the active compounds and activity mechanisms quickly and achieved the change from experience-based medicine to evidence-based medicine (Zhou et al., 2020), which supplied novel guidance, strategy, thinking, and research for TCM. Nevertheless, our data did not yet allow us to know what behaviors of MVO were *in vivo* and whether MVO protected the hypertension-related vascular dysfunction and other complications. Hence, *in vivo* experiments should be performed to study the underlying mechanism of MVO-induced vasorelaxation for further investigation.

## 6 Conclusion

In the present study, the results of GC-MS revealed that 29 compounds were found in MVO and α-, β-, and γ-eudesmol occupied a large proportion in MVO. The network pharmacology indicated that a total of seven ingredients, of which the top three were beta-caryophyllene, palmitic acid, and (+)-β-selinene, were more likely to show therapeutic effects against hypertension *via* multiple targets and multi-pathways. Moreover, the results highlighted the idea that MVO could regulate the expression levels of targets, especially for TNF, CHRM1, ACE, IL10, PTGS2, REN, and F2, as well as neuroactive ligand–receptor interaction, the calcium signaling pathway, and PI3K-Akt signaling pathway to treat hypertension. Similarly, the vasorelaxant effects and mechanisms of MVO were identified in aortic rings, mainly including the PI3K-Akt-NO pathway, K_V_ pathway as well as Ca^2+^ channel. The present study not only reported the vasorelaxant activity and the underlying mechanisms of MVO but also provided a useful strategy for TCM because of the combination of network pharmacology and experiments *in vitro*.

## Data Availability

The original contributions presented in the study are included in the article/Supplementary Material, further inquiries can be directed to the corresponding authors.

## Abbreviations

4-AP, 4-aminopyridione; +E, endothelium-intact; -E, Endothelium-denuded; Ach, acetylcholine chloride; CCVDs, cardiac-cerebral vascular diseases; C-T, compound-target; C-T-D, compound-target-disease; C-T-P, compound-target-pathway; Emax, the maximum relaxant magnitude; Et, the real-time relaxant magnitude; GC-MS, gas chromatography-mass spectrometry; Gli, glibenclamide; GO, gene ontology; Indo, Indo-methacin; KATP, ATP-sensitive K+ channels; KCa^2+^, Calcium-activated K+ channels; KEGG, Kyoto Encyclopedia of Genes and the Genomes; KIR, Inward rectifier K+ channels; KV, Voltage-dependent K+ channels; L-NAME, N-nitro-L-arginine methyl ester; MB, methylene blue; MVO, Magnolia volatile oil; PHE, Phenylephrine; PPI, protein-protein interaction; ROCC, receptor-operated Ca^2+^channel; TCM, traditional Chinese medicines; TEA, tetraethyl-ammonium; VDCC, voltage-dependent Ca^2+^ channel.
